# Optimization of Bioink Formulations and Bioprinting Conditions for Enhanced Cell Viability in Particle-Containing Constructs

**DOI:** 10.3390/polym18162021

**Published:** 2026-08-20

**Authors:** Fiona Ye Rojo Acero, Daniel F. de Castro Hernández, María Lisseth Flores-Cedillo, Juan José Uriarte, Ainhoa Herrero, Raquel Villa, Luis M. Rodríguez-Lorenzo

**Affiliations:** 1 Bio2, Biomateriales y Bioimpresión, ICTP-CSIC, 28006 Madrid, Spain; f.rojoacero@gmail.com (F.Y.R.A.); dcastroh@alumnos.nebrija.es (D.F.d.C.H.); ainhoaherrero@protonmail.com (A.H.); villaplazaraquel@gmail.com (R.V.); 2 ARIES Research Group, Escuela Politécnica Superior, Universidad Nebrija, 28015 Madrid, Spain; juriarte@nebrija.es; 3 Tecnológico Nacional de México/Instituto Tecnológico Superior de San Luis Potosí Capital, San Luis Potosí 78421, Mexico; maria.flores@tecsuperiorslp.edu.mx

**Keywords:** bioprinting, bioink, particle-containing bioinks, pseudoplastic fluid, shear thinning fluids, tissue engineering, bioprinting design, hydroxyapatite, regenerative medicine, particle-containing constructs

## Abstract

Extrusion-based bioprinting imposes stringent mechanical constraints on bioink formulations, yet the rheological parameters governing cell survival during the printing process are rarely reported in a standardized way, limiting cross-study comparison. In this work, we systematically characterized the viscoelastic properties of alginate/methylcellulose bioinks incorporating strontium-enriched hydroxyapatite (Sr-OHAp) particles and Poloxamer 188, and assessed their effect on PANC-1 cell viability in bioprinted constructs. The power law consistency index K and pseudoplasticity index *n* were used as quantitative descriptors of bioink behavior. Addition of Poloxamer 188 reduced K by 52.1% in particle-free inks and by 64.6% in particle-containing inks, while n remained largely unchanged (≤2% variation), indicating that particles selectively modulate consistency without compromising shear-thinning behavior. On day 1, bioprinted constructs showed lower cell viability than cell-seeded scaffolds (53.9–58.9% vs. 96.7%); however, constructs containing Sr-OHAp (B3) displayed progressive recovery, reaching 84.0% viability by day 7, compared to 72.9% for particle-free bioinks (B1). These results demonstrate that Sr-OHAp particles act as rheological sensitizers that reduce extrusion-induced shear stress while simultaneously promoting long-term cell recovery, likely through their bioactive surface chemistry. We propose that systematic reporting of K and n indices should become standard practice in bioprinting studies to enable rational bioink design and consistent knowledge accumulation across the field.

## 1. Introduction

The success of bioprinting technology as a technology of choice in tissue engineering or clinical applications will strongly depend on the capacity to develop appropriate bioinks for each specific application and/or printing conditions. The properties of the bioink and the resulting construct are not independent at any stage of the process. From the bioink in a low-viscosity state to the engineered construct and finally the functional implant or model, these characteristics are interrelated and can influence each other throughout physical and chemical transformations [1]. Therefore, understanding these properties is crucial for ensuring successful implementation [2]. Building on previous findings [3,4], in this study, we aimed to investigate the relevance of using particle-containing bioinks on the biological response and printing capacity of the prepared bioinks and the features of the resulting constructs.

The effect of particle content in bioink features, such as printability, shape fidelity, long-term stability, and cell viability, are an open field of research [5,6,7,8]. Micro- and nanofillers are employed in numerous studies to enhance the performance of bioink while maintaining the physicochemical properties of the polymeric material that constitutes the majority of the bioink [9]. Reinforcement with particles or fibers is a classical method for modulating the mechanical performance of polymeric matrices [10,11]. A commonly expressed limitation of bioprinting in tissue engineering is the discrepancy between the mechanical properties of bioprinted constructs and those of natural tissues. Consequently, the reinforcement of continuous matrix bioinks with particles is being investigated [12]. The most frequent type of particles used in particle-containing bioinks are from the hydroxyapatite or bioglass family [7,13,14,15]. However, the criteria for designing particle-containing bioinks should include not only mechanical performance but also biological stimuli [9].

Hydroxyapatites (OHAps) are a family of compounds that promote fibroblast activity and collagen deposition; therefore, their use has been extended from hard to soft tissues, and studies related to soft tissue augmentation or skin models have appeared more frequently in the literature in recent years [16,17,18]. In addition, OHAps may act as drug/growth factor carriers, leveraging their porous structure and surface chemistry, such as Zn^2+^, Cu^2+^, and Sr^2+,^ for sustained delivery of antibiotics and bioactive molecules (e.g., vancomycin or VEGF) or ion release, which synergizes antimicrobial potency with pro-angiogenic and anti-inflammatory effects [19,20]. OHAps also integrate effectively with biopolymers to form composites that enhance mechanical stability and cellular response. These innovations together have shown promise for further applications as in *cancer* theranostics and cancer therapy [21,22]. The introduction of strontium into hydroxyapatite alters its crystallinity, limits particle growth/aggregation, and modifies the dissolution rate of calcium phosphate nanoparticles, while also changing their net surface charge. Sr-OHAp nanoparticles have shown long-term physiological stability, efficient endosomal escape, and have been used as vectors for gene delivery showing optimal cargo delivery within cells [20,23]. For these reasons Sr-OHAp were selected in this work, with a view to their future application in PANC-1-based tumor modelling and gene therapy.

Alginate remains a favored component in bioink compositions because ionic crosslinking with calcium ions is preferred for biomedical applications due to the mild conditions that can be used and the simplicity and kinetics of ionotropic gelation, which avoids potentially toxic reagents or UV radiation [2]. Poloxamers are commonly incorporated into bioink formulations owing to their unique thermoresponsive and rheological properties, which are highly beneficial for bioprinting applications. Specifically, poloxamers exhibit reversible sol–gel transitions in response to temperature changes, allowing the bioink to remain fluid at lower temperatures for easy extrusion and to rapidly gel at physiological temperatures to maintain structural fidelity after printing [24]. This thermogelling behavior enhances printability by providing shear-thinning properties during extrusion and rapid solidification after deposition, which supports the shape retention and mechanical stability of printed constructs. Additionally, poloxamers can improve the homogeneity and dispersion of other bioink components, such as cells and particles, contributing to a more uniform microenvironment for cell growth. However, the concentration of poloxamer should be kept low and photo-polymerization avoided to diminish the risk of cell toxicity [25].

The objective of this study was to investigate the effects of incorporating strontium-enriched hydroxyapatite particles and F188 poloxamer on the printing capacity of PANC-1 containing alginate bioinks and cellular viability of bioprinted constructs to understand the processes of optimizing bioink compositions for building constructs for specific applications.

## 2. Materials and Methods

### 2.1. Components of the Inks and Bioinks

Alginic acid sodium salt from brown algae (SA) (Mw: 8945 g/mol, mannuronate/guluronate ratio of 0.63, Sigma, Saint-Quentin-Fallavier, France) [26], methylcellulose (MC) (viscosity 4000 cP, M0512-500 G), and Poloxamer 188 PRO P4894) were purchased from Sigma-Aldrich (Madrid, Spain). Sr^2+^-enriched hydroxyapatite particles (Sr-OHAp) were synthesized and characterized as described elsewhere [27]. PANC-1 cells, an epithelioid carcinoma cell line derived from the human pancreas [28], were provided by ATCC (frozen, CRL-1469, tissue: Pancreas; Duct) from the human cell culture collection (https://www.atcc.org/).

### 2.2. Cell Culture

PANC-1 cell lines were cultured in RPMI (Gibco/Invitrogen, Carlsbad, CA, USA) supplemented with 10% fetal bovine serum (FBS; Invitrogen) and 50 units/mL penicillin/streptomycin (Invitrogen) and kept in an incubator at 5% CO_2_ and 37 °C. https://genome.ucsc.edu/ENCODE/protocols/cell/human/PANC-1_Myers_protocol.pdf. accessed on 19 August 2026.

### 2.3. Inks and Bioinks Preparation

Six inks and two bioinks were prepared using the following concentrations: alginate 3% *w*/*v*, methylcellulose 5% *w*/*v*, poloxamer 1.25% *w*/*v* (bioink 1); alginate 3% *w*/*v*, methylcellulose 5% *w*/*v*, poloxamer 1.25% *w*/*v* using RPMI as solvent where cells will be incorporated after printing (ink 2a); alginate 3% *w*/*v*, methylcellulose 5% *w*/*v*, poloxamer 1.25% *w*/*v* (ink 2b); alginate 3% *w*/*v*, methylcellulose 5% *w*/*v* (ink 2c); alginate 3% *w*/*v*, methylcellulose 5%, *w*/*v* poloxamer 1%, Sr-OHAp 3% (bioink 3); alginate 3% *w*/*v*, methylcellulose 5% *w*/*v*, poloxamer 1.25% *w*/*v* where cells were incorporated after printing (ink 4a); alginate 3% *w*/*v*, methylcellulose 5% *w*/*v*, poloxamer 1.25% *w*/*v* (ink 4b) and alginate 3% *w*/*v*, methylcellulose 5% *w*/*v* (ink 4c).

All inks and bioinks were prepared to a final volume of 10 mL. For bioinks, PANC-1 cells were first counted and resuspended in poloxamer solution in RPMI culture medium. Separately, SA and MC were dissolved in RPMI under stirring until homogeneous; MC homogeneity was achieved after overnight rest at 4 °C. MC gel was then added to the SA gel and mixed with a spatula until homogeneous. Finally, the cell-poloxamer suspension was gently incorporated into the hydrogel blend. Inks were prepared following the same protocol; in this case, the equivalent volume of RPMI or RPMI/poloxamer was added in place of the cell-poloxamer suspension. All inks and bioinks were prepared at least 9 times to ensure reproducibility.

### 2.4. Rheological Characterization

Rheological measurements were performed using an AR-G2 rheometer (TA Instruments, New Castle, DE, USA) with a sand-blasted parallel-plate geometry (diameter: 25 mm). All the experiments were replicated at least with three different preparations for each composition. Samples were loaded onto the preheated rheometer Peltier plate at 25 °C using a syringe up to a height of 1000 µm and measured after 30 s of stabilization.

A shear rate sweep ranging from 0.1 to 300 s^−1^ was performed. In addition, the yield stress point was calculated as the intersection between viscosity and shear stress [29]. The rotational recovery of each ink was measured by subjecting it to a shear rate of 1 s^−1^ for 60 s, followed by a shear rate of 100 s^−1^ for 30 s. This process was performed twice at the maximum shear rate and three times at the minimum shear rate to evaluate the materials behavior in the recovery process. The linear viscoelastic region (LVR) was determined using an oscillatory stress sweep test at 1 Hz to select a 1% strain at 25 °C and perform a frequency sweep between 10^−2^ and 100 Hz. After crosslinking with 1.5% CaCl_2_, a time test was performed at 25 °C for 10 min, followed by 20 min at 37 °C. Shear-thinning fluids are also characterized by the Ostwald–de Waele power law model. The model is expressed as follows:

(1) $$\eta ={K\dot{\gamma }}^{\;n-1}$$

(2) $$\tau ={K\dot{\gamma }}^{\;n}$$

where ɳ is the viscosity, K is the flow consistency index, $\dot{\gamma }$ is the shear rate, and n is the flow behavior index. The shear stress versus shear rate was plotted in Origin, and an allometric model was used to calculate the index [30].

### 2.5. 3D Printing and Bioprinting

Inks and cell-laden bioinks were loaded into a 3 mL cartridge and stored at room temperature (RT) to stabilize the rheological properties of the bioink. A pneumatic extrusion Inkredible 3D printer (Cellink, Gothenburg, Sweden) was used to dispense the hydrogel bioink through a 0.60, 0.41 and 0.25 mm; 20, 22 and 25 G; conical nozzles. Cylindrical constructs of diameter 15 mm were printed into 6-well cell culture plates with a strand width of 1.8 mm, creating 3D stacking openings of 1.3 mm (and an infill extrusion width of 0.48 mm). Printed hydrogels were crosslinked for 20 min in a bath with 1.5% *w*/*v* CaCl_2_, after which the crosslinker was removed and replaced with fresh medium. Finally, the constructs were incubated at 37 °C and 5% CO_2_. All the compositions were either printed or bioprinted at least three times.

The measured values of the K and n indices of the bioinks were used to calculate the shear rate along the needle radii using Equations (3) and (4) for the needle tip. The calculated values of the shear rate were then plotted against the radii of the needle. Then, Equations (1) and (2) were used to calculate the values of viscosity and shear stress. The calculated shear stress and shear rate were plotted against one another as displayed below.

(3) $$\dot{y_{1}}=(\frac{V_{1}R_{1}^{2}}{\left(\frac{n}{3n+1}\right)\left(R_{1}^{\frac{3n+1}{n}}\right)})\sqrt[{n}]{r}$$

(4) $$\dot{y_{2}}=\left(\frac{V_{2}R_{2}^{2}}{\left(\frac{n}{3n+1})(R_{2}^{\frac{3n+1}{n}}\right)}\right)\sqrt[{n}]{r}$$

where $\dot{\gamma }$ is the shear rate, V the dispensing speed, *R*_1_ the cartridge radius and *R*_2_ is the radius at the tip, and *r* the ratio at the calculation point.

### 2.6. Characterization of the 3D Printed Scaffolds and Constructs

A gravimetric test was used to evaluate the swelling behavior of the printed scaffolds [31]. The printed scaffolds were placed and submerged in cell culture medium in a 6-well plate culture and incubated at 37 °C for different time points. The initial dry weight (*wd*) of each scaffold was measured after eliminating excess cell culture medium with a paper towel. The swollen weight percentage (*S*) was measured at different times (1, 2, 4, 8, 19, and 24 h). 4 replicates per composition were used per composition. Equation (5) was used to determine the swelling behavior.

(5) $$S=\left(\frac{w_{s}-w_{d}}{w_{d}}\right)\times 100\%$$

An Axio Lab.A1 Zeiss optical microscope (Zeiss, Oberkochen, Germany), a Field emission scanning electron microscope APREO 2 (Thermo Fisher, Waltham, MA, USA), equipped with secondary and backscattered electron detectors, both in chamber and in-lens STEM modes, EDX, windowless EDX, and a CRYO-FEGSEM microscopy unit operating in high vacuum mode from MNCN-CSIC, Madrid were used to obtain optical micrographs, SEM micrographs and cryomicrographs.

### 2.7. Viability Assay on Cell-Laden Constructs

Cellular survival and proliferation were assessed using a live/dead viability kit. Calcein AM (0.5 µL) and ethidium homodimer-1 (2.0 µL) were dissolved in 997.5 µL PBS, added to the samples, and incubated for 30 min in the dark at 37 °C in a humidified atmosphere with 5% CO_2_. (EthD-1) (ThermoFisher Scientific #L3224, Karlsruhe, Germany) according to the manufacturer protocol. Constructs are imaged with a Leica Paula and a Leica TCS SPE, Wetzlar, Germany, from MNCN-CSIC, Madrid. A total of 3 specimens were used per preparation.

### 2.8. Statistics

Three repetitions were conducted to verify the reproducibility of the results obtained in the rheology and cytocompatibility test. A one-way ANOVA was performed. Tukey’s post hoc test was applied. Statistical differences were assumed at *p* < 0.05. All analyses were performed using STATA/SE, StataCorp LLC Statistics/Data Analysis (Special Edition, College Station, TX, USA).

## 3. Results

Table 1 presents the ink and bioink compositions selected for this study. The base composition (3% alginate/9% methylcellulose) was adopted from a previous work [3]. From this starting point, methylcellulose concentration was varied (3%, 5%, and 9% *w*/*v*) while alginate was kept fixed, and hydroxyapatite was added at 0–5% *w*/*v*. The final compositions were selected based on preliminary printability tests evaluating filament integrity and structural stability.

The chemical composition of Sr-OHAp was Ca_8.6_Sr_1.4_(PO_4_)_6_(OH)_2_. The particles formed agglomerates with 1.57, 5.9, and 14.96 µm for d10, d50, and d90, respectively, values compatible with 0.25 mm inner diameter nozzles.

### 3.1. Characterization of Inks and Bioinks

**Figure 1:**
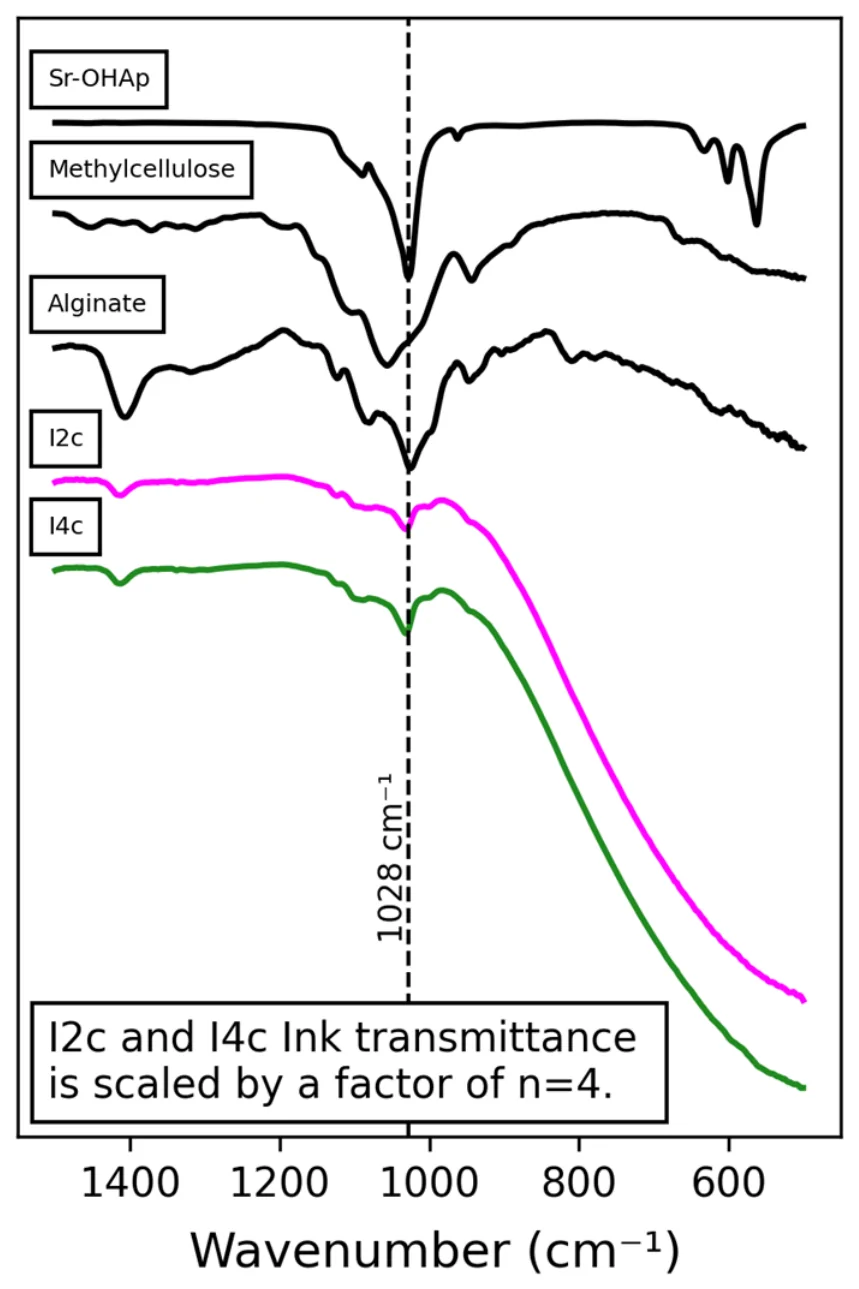
Infrared spectra of inks and their components. The spectra of the inks displayed bands corresponding to both the hydroxyapatite and polysaccharide components, indicating the integration of all components within the material. Band assignment is detailed in the main text.

The IR spectra of I2_c_ and I4_c_ and their individual components are shown in Figure 1. The spectrum of Sr-OHAp shows bands at 1028 and 1065 cm^−1^ that correspond to the υ_3_ symmetric and asymmetric bands of phosphate in the apatite environment, a band at 961 cm^−1^ that can be assigned to the υ_1_ band of phosphate, bands at 530, 575 due to υ_4_ bands of the phosphate, and a band at 630 cm^−1^ that can be assigned to the OH^−^ group [32], suggesting some degree of crystallinity [33]. The spectrum of methylcellulose displays peaks at 1452 and 1373 cm^−1^ related to δ_C-H_; weak peaks are observed at 1312 cm^−1^ and 1192 cm^−1^. The first is associated with the δ_OH_ group, and the second is associated with υ_C-O_. These peaks suggest incomplete methylation and the presence of residual OH^−^ groups. The strong peak at 1052 cm^−1^ is associated with δ_C-O-C_ of the methylated rings, and the peak at 946 assigned to υ_C-O_ [34]. The spectrum of alginate displays a band at 1410 cm^−1^ that can be associated with υ_s COO−_, while υ_C-O_ bands are detected at 1320 cm^−1^ and 1290 cm^−1^. Bands at 1022 and 1081 cm^−1^ were identified for υ_C–C_ and υ_C–O_ from the pyranose ring in alginate. Finally, δ_C-O_ from uronic acid residues was observed at 949 cm^−1^, and δ_C1-H_ from β-mannuronic acid residues was observed at 878 cm^−1^ [26]. The spectra of the inks displayed bands corresponding to both the hydroxyapatite and polysaccharide components, indicating the integration of all components within the material. The presence of the strongest band from Sr-OHAp at 1028 cm^−1^ and the shift in the υ_sCOO−_ band of alginate can be highlighted.

### 3.2. Rheological Properties of Inks and Bioinks

**Figure 2:**
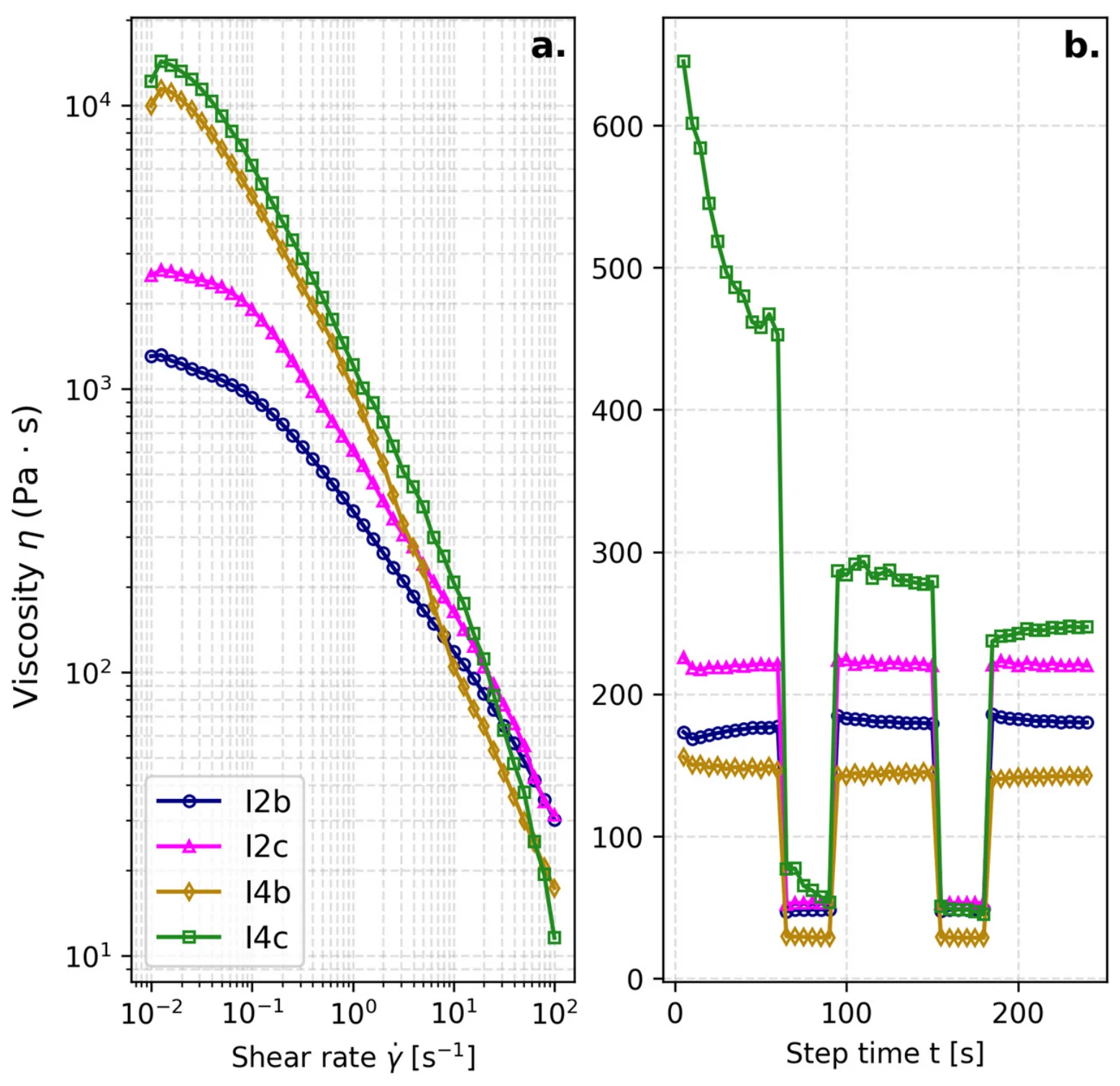
Rotational rheological measurements performed using an AR-G2 rheometer with a 25 mm diameter sand-blasted parallel-plate geometry. (**a**) Viscosity versus shear rate for poloxamer-free and poloxamer-containing inks. (**b**) Recovery test for the same compositions. The flow recovery of each ink was measured by subjecting it to a shear rate of 1 s^−1^ for 60 s, followed by a shear rate of 100 s^−1^ for 30 s. Other experimental conditions are described in Section 2.4.

Rotational measurements were used to test the influence of adding poloxamer and particles to the ink composition. All the compositions tested exhibited shear-thinning behavior, as shown in Figure 2a. Cells to be incorporated into the compositions to prepare bioinks require the addition of a protective poloxamer component. The addition of this component produced less viscous inks in the steady state and a lower decrease in viscosity in the flowing mode (lower slope on the graph after the yielding stress point). The ceramic particles affect the viscosity of the inks in the steady state more significantly for the poloxamer-containing compositions than for poloxamer-free compositions, and they also increase the value of the yielding stress point. The recovery test displayed in Figure 2b indicates that the presence of ceramic particles without the poloxamer decreases the recovery capacity of the inks. Inks I2b, I2c and I4b, produced a 100% recovery of the viscosity in the steady state after the flow cycle with no delay.

**Figure 3:**
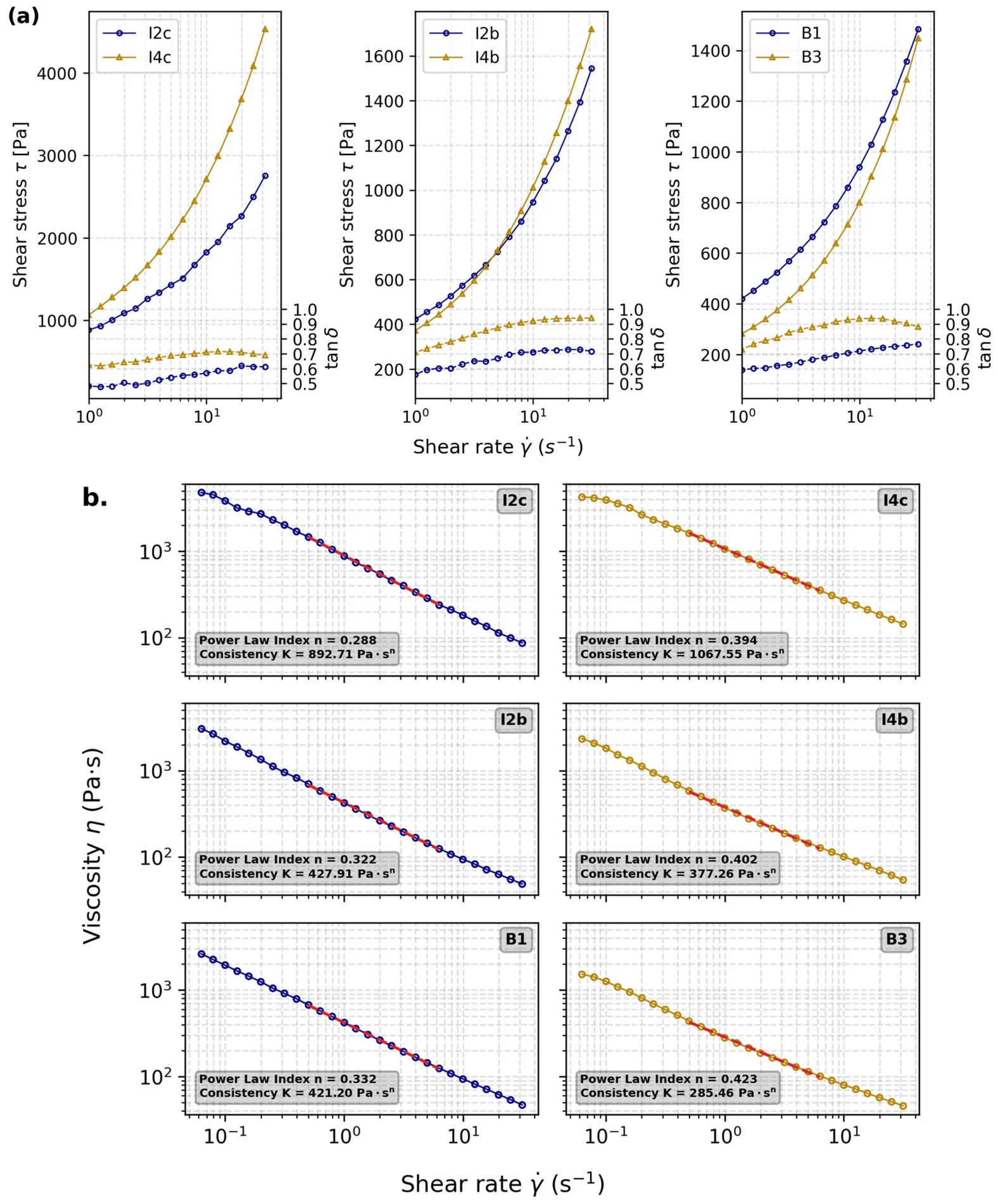
(**a**) Shear stress versus shear rate and tan δ for I2, I4, B1 and B3. (**b**) Complex viscosity as a function of frequency, obtained via the Cox–Merz rule. Power law indices K and n were calculated using an allometric model.

The viscoelastic behavior of inks and PANC-1 containing bioinks were evaluated through oscillatory measurements. The shear stress versus shear rate results are shown in Figure 3a, and complex viscosities obtained via the Cox–Merz rule are shown in Figure 3b. The dependence of the elastic modulus on the frequency variation is characteristic of entangled networks [35]. For both I2 and I4, the incorporation of the poloxamer component produced a shift in the power law toward a less pseudoplastic behavior. The incorporation of PANC-1 cells also resulted, for both compositions, in a less pronounced pseudoplastic behavior of the bioinks in comparison with the reference inks. The results for tan δ are also shown in Figure 3a. The curves show the prevalence of the G″ over G′ for all compositions. The incorporation of poloxamer increased the tanδ value for both compositions, but the incorporation of PANC-1 cells had a less marked effect.

### 3.3. Printability and Filament Quality

**Figure 4:**
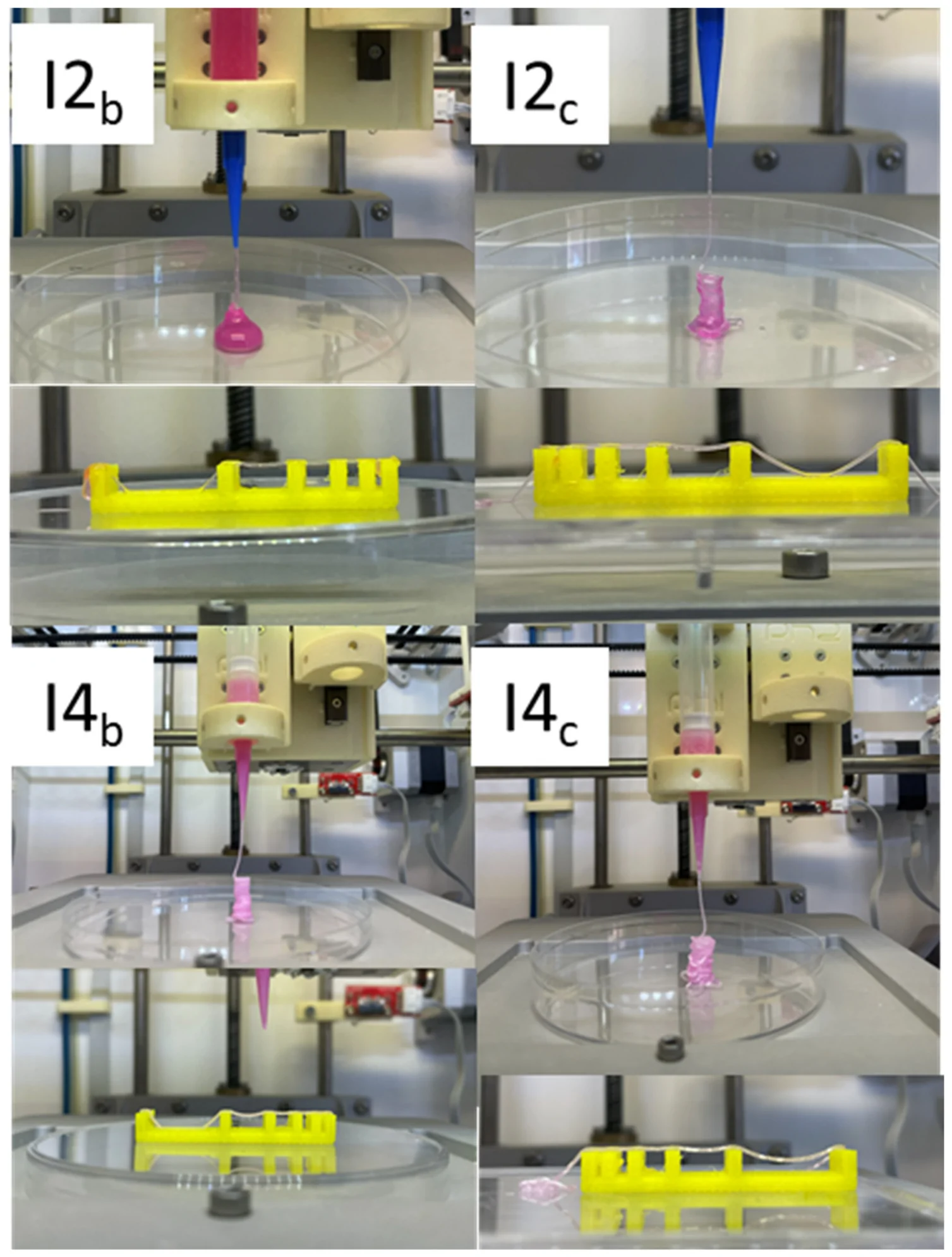
Extruded filaments and collapsing test for inks I2b, I2c, I4b and I4c. A pneumatic extrusion Inkredible 3D printer was used to dispense the hydrogel inks. The images show examples of dispensing through conical nozzles of 0.60 mm (20 G, pink) and 0.41 mm (22 G, blue).

The visual aspect of the extruded filaments and the collapsing test can be observed in Figure 4. All filaments appeared continuous and uniform in thickness, indicating consistent extrusion and deposition. The surface texture appeared smooth, with no visible breaks or irregularities, implying good material flow and stability. The incorporation of poloxamer into inks 2b and 4b resulted in a greater restriction on the maximum bridge width that could be built in the constructs. Rapid solidification through crosslinking appears to be necessary from the piling of the layers, particularly for particle-free inks 2b and 2c.

### 3.4. Morphology of Bioprinted Constructs

**Figure 5:**
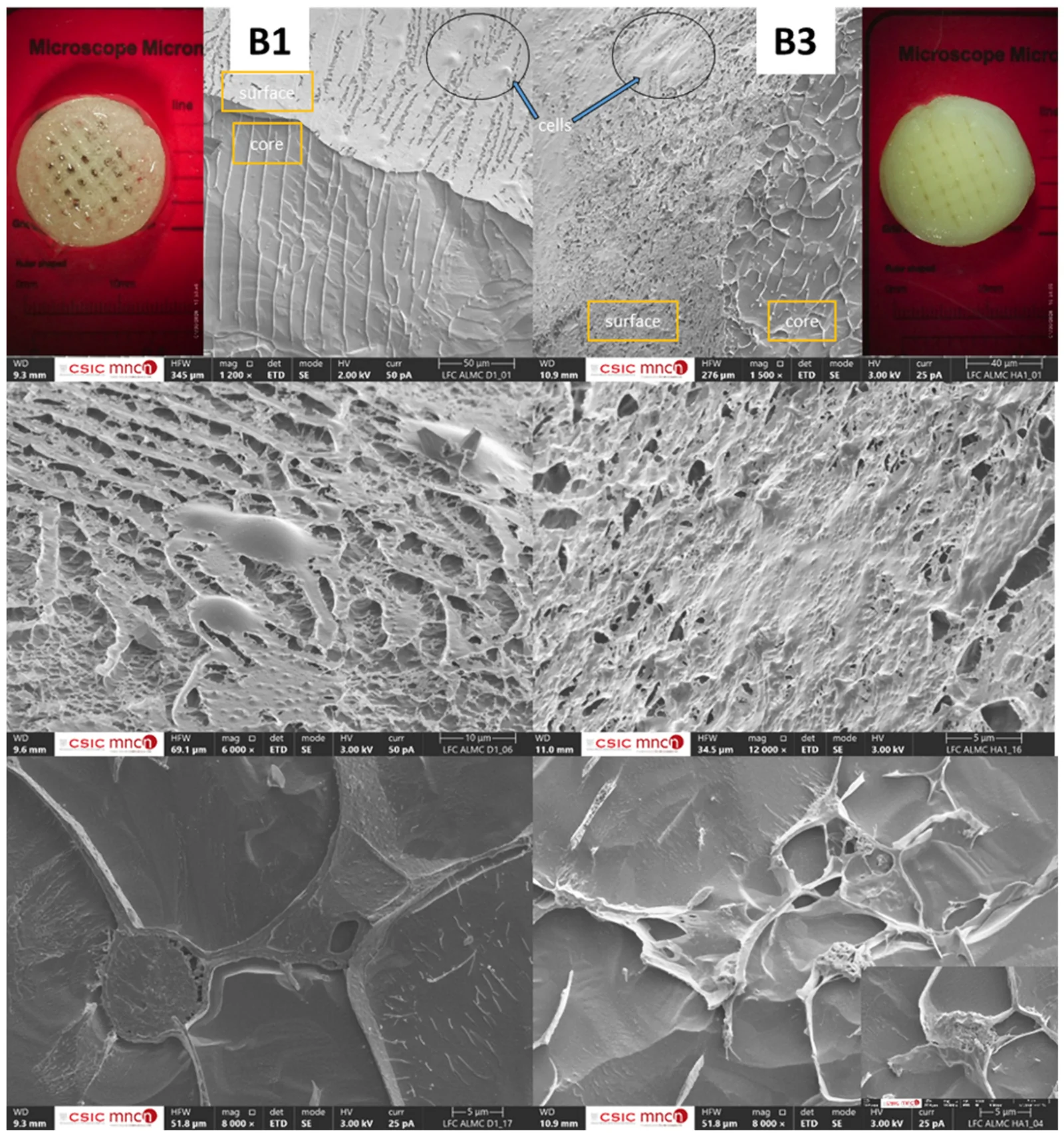
Optical microscopy photographs (Axio Lab.A1, Zeiss) and cryomicroscopy images (CRYO-FEGSEM, MNCN-CSIC, high vacuum mode) of bioprinted constructs from bioinks B1 and B3. Cryomicroscopy images reveal the micro-scale arrangement and morphology of the components after freezing, preserving their native hydrated state.

Figure 5 displays the aspects of the bioprinted constructs, together with cryomicroscopy images of the constructs from bioinks B1 and B3. Cryomicroscopy images reveal the micro-scale arrangement and morphology of the components after freezing, preserving their native hydrated state. The top-left image of the construct built with B1 shows both surface and internal features, where the parallel lines in the core correspond to layered deposition during bioprinting, and some cells are visible on the surface. In contrast, the construct from B3 exhibited greater diffusion of bioprinting layers within the core and a higher density of cells attached to the surface. The central images illustrate the differences in cell attachment, with cells on the B3 construct surface spreading more extensively than those on the B1 construct. The bottom line images from the core region show cells integrated within the construct adhering to solid filament-like lines. Additionally, hydroxyapatite particles are visible embedded within the polymer matrix of the B3 construct, surrounded by biological material, as magnified in the inset.

### 3.5. Scaffold Degradation and Cell Viability

**Figure 6:**
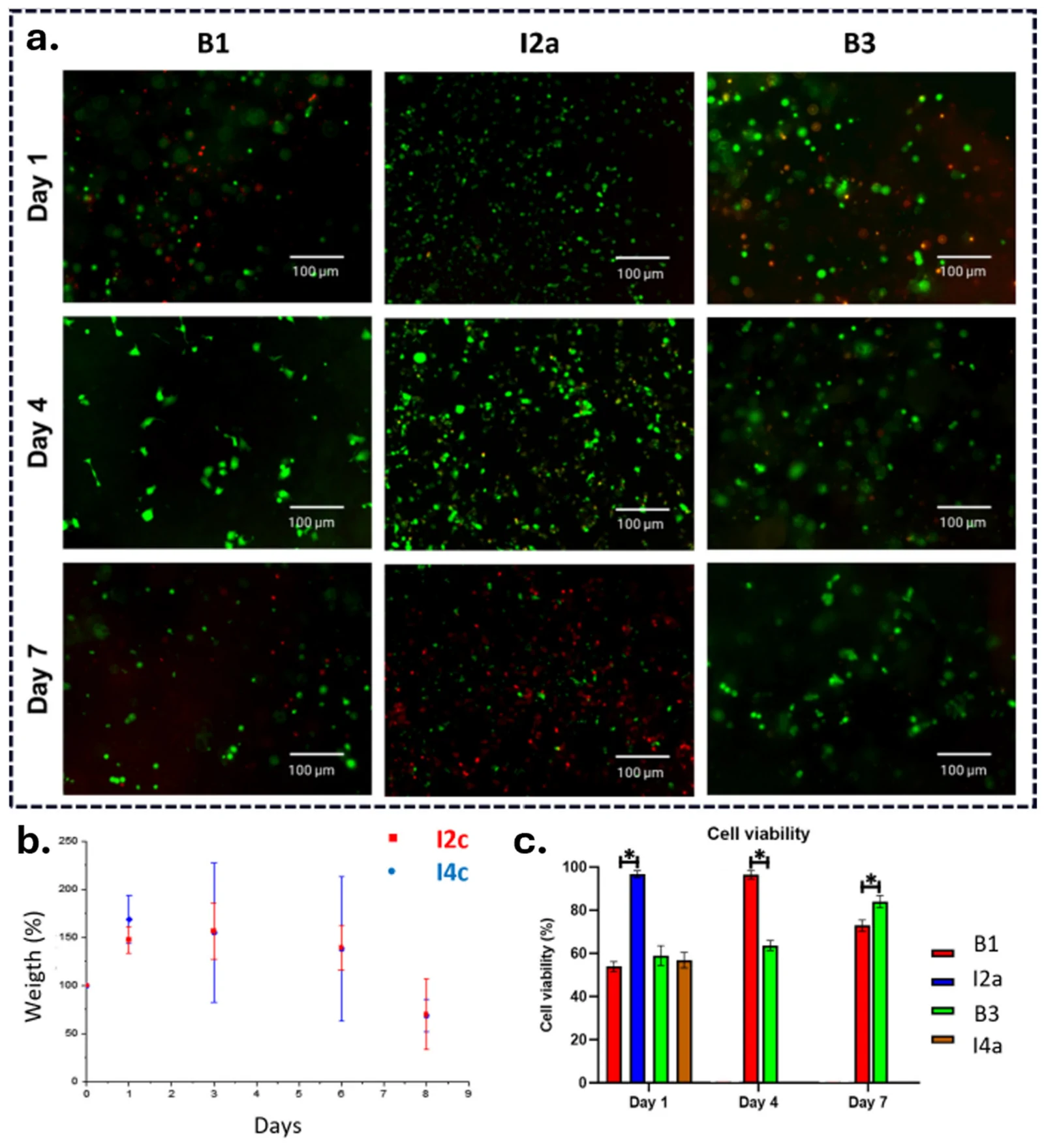
(**a**) Fluorescence images of bioprinted constructs on days 1, 4, and 7 (calcein AM. Green for live cells, EtD-1, red for dead cells) taken with a Leica TCS SPE confocal microscope; (**b**) scaffold weight loss during degradation; (**c**) cell viability quantified over 7 days; Assay conditions are described in Section 2.6 and Section 2.7; * *p* < 0.05.

The degradation is shown in the lower left of Figure 6. It can be appreciated that the incorporation of Sr-OHAp does not significantly modify the swelling and degradation behavior of the tested scaffolds. An initial swelling was observed that lasted up to 6 days, followed by a sharp weight loss between days 6 and 8. The main body of Figure 6 displays the cell viability ratio after printing, bioprinting, and its temporal evolution. PANC-1 viability on cell-laden scaffolds was assessed on day 1 only, as the primary focus of this work was the effect of printing-induced pressure on cell viability rather than long-term culture outcomes. On the initial day of observation, I2a displayed the highest cell viability at 96.72%, compared to B1, B3, and I4a, which showed viabilities of 53.86%, 58.94%, and 56.84%, respectively. By day 4, the cell viability increased for B1 and B3, reaching 96.40% and 63.65%, respectively. On day 7, all the bioprinted compositions maintained cell viability above 70%. However, B1 experienced a decline in viability to 72.86%, whereas B3 displayed an increase in viability to 83.96%. This temporal evolution reflects the influence of the composition and stress experienced by the cells during the extrusion process. Constructs made by seeding after extrusion with no Sr-OHAp particles show initially a higher viability but the viability decreases with time whereas bioprinted construct containing Sr-OHAp particles exhibits a progressive improvement in cell viability.

## 4. Discussion

3D bioprinting enables the fabrication of structurally well-defined objects, allowing the design of cell distribution, favoring cell viability and functionality, and enabling the creation of complex and heterogeneous architectures that exhibit a broad spectrum of biological and mechanical properties. These characteristics have led to a surge in studies utilizing this technology for tissue engineering and modeling applications [36,37,38,39,40]. Bioprinting involves combining cells with biomaterials to form a bioink prior to the 3D printing process. This biofabrication method imposes stringent constraints on the properties of the biomaterials that can be used. These biomaterials should be compatible with cell viability and function throughout the entire process [41].

A key requirement is that bioinks need to be made of shear-thinning materials that induce lower shear stress on cells during the extrusion process through the tip to reduce cell damage [42]. Thus, a common additional precaution in bioprinting is to soak the cells in a protective component before mixing them with the rest of the bioink materials. Among the potential cell-protective components, poloxamers, plasma, and leukocyte- and platelet-rich fibrin (L-PRF) can be selected. Plasma is one of the most used [3,43,44] although its behavior is rather Newtonian. L-PRF is a shear thinning material that offers an additional biological advantage through the sustained release of bioactive molecules [45]. However, to simplify the study, a poloxamer was selected. As stated in the introduction, poloxamers provide shear-thinning properties and have been used in cell cultures and drug delivery for a long time [25]. From the cell viability studies presented in Figure 6c, it can be observed that for particle-free compositions, there is a significant difference in cell viability for cells seeded after printing in comparison with cells embedded for bioprinting I2a vs. B1; however, for particle-containing bioinks, there is no significant difference I4a vs. B3. This result can be related to the viscoelastic changes that the introduction of particles induces in the bioinks as it will be explained later.

The results presented in Figure 3 indicate that for particle-free compositions, the presence of poloxamer in I2b reduces the consistency index K by 52.1% and increases the pseudoplasticity index n by 10.55%. In contrast, in the presence of particles, I4b, the presence of poloxamer only significantly reduced K (64.59%), whereas the increase in the n index was much smaller (1.99%). In bioinks, when cells are incorporated, in the particle-free composition with poloxamer, B1, K and n are barely modified in comparison with I2b, whereas for bioink B3, the consistency index K is further reduced beyond what is achieved with poloxamer alone; specifically, the presence of cells reduces consistency by an additional 8.62% compared to the poloxamer alone. The n index also shows a comparatively larger change than I2b, but in absolute terms, it remains small. The presence of particles appears to act as a sensitizer of the consistency index K; in the presence of particles within the ink and bioink compositions. The influence of particle concentration in inks for extrusion-based bioprinting has been previously analyzed in terms of its effects on ink viscosity at low and high shear stress, concluding that a higher particle content increases the initial pressure required for extrusion compared to inks with lower particle content, but excessively high particle contents may undermine the printing fidelity of the printed device [46,47]. However, very few manuscripts [2,3] actually quantify and report how particle content affects the viscoelastic properties of the ink, as reflected in the K and n indices of the power law described in Equation (1). This is particularly relevant for the rational design of bioinks, since both the power law model and the alternative Herschel–Bulkley model rely on simplifications and assumptions that require experimental validation [47]. In addition, these parameters govern the shear stress experienced by cells during the printing process and, consequently, cell viability, as discussed below. Thus, their relevance extends beyond printing fidelity, becoming a fundamental parameter for cell viability in 3D bioprinted constructs.

K decreases significantly when poloxamer is added, while n is reduced to a significantly lesser extent than that of analog particle-free compositions. Consequently, the incorporation of particles produces inks and bioinks with lower consistency, but the sacrifice of pseudoplasticity is minimal. These differences in viscoelastic behavior must be related to the different cell viability values discussed above, as they mitigate the stress suffered by the cells during the printing process, suggesting that the incorporation of particles in bioink compositions may favor long-term cell fate.

Another consequence of introducing particles within a polymeric matrix is the introduction of a new degree of freedom for bioink design. The pseudoplastic parameter n is an essential parameter in designing inks with low shear stress [48,49]. The advantage is that the introduction of bioactive particles such as the ones used in this work enable tailoring of the biological response of cells by favoring attachment and proliferation to the usually very hydrophilic compositions of bioinks [5,50,51] avoiding the need for complicated chemical modifications [52] or crosslinking additives [53] that may modulate the requested biological response [54] or become cytotoxic [55]. In the current work, the aim of reducing shear stress on cells while going through the tip has been achieved by adding Sr-OHAp particles and poloxamer F188 to the combination of PANC-1, alginate and methylcellulose.

**Figure 7:**
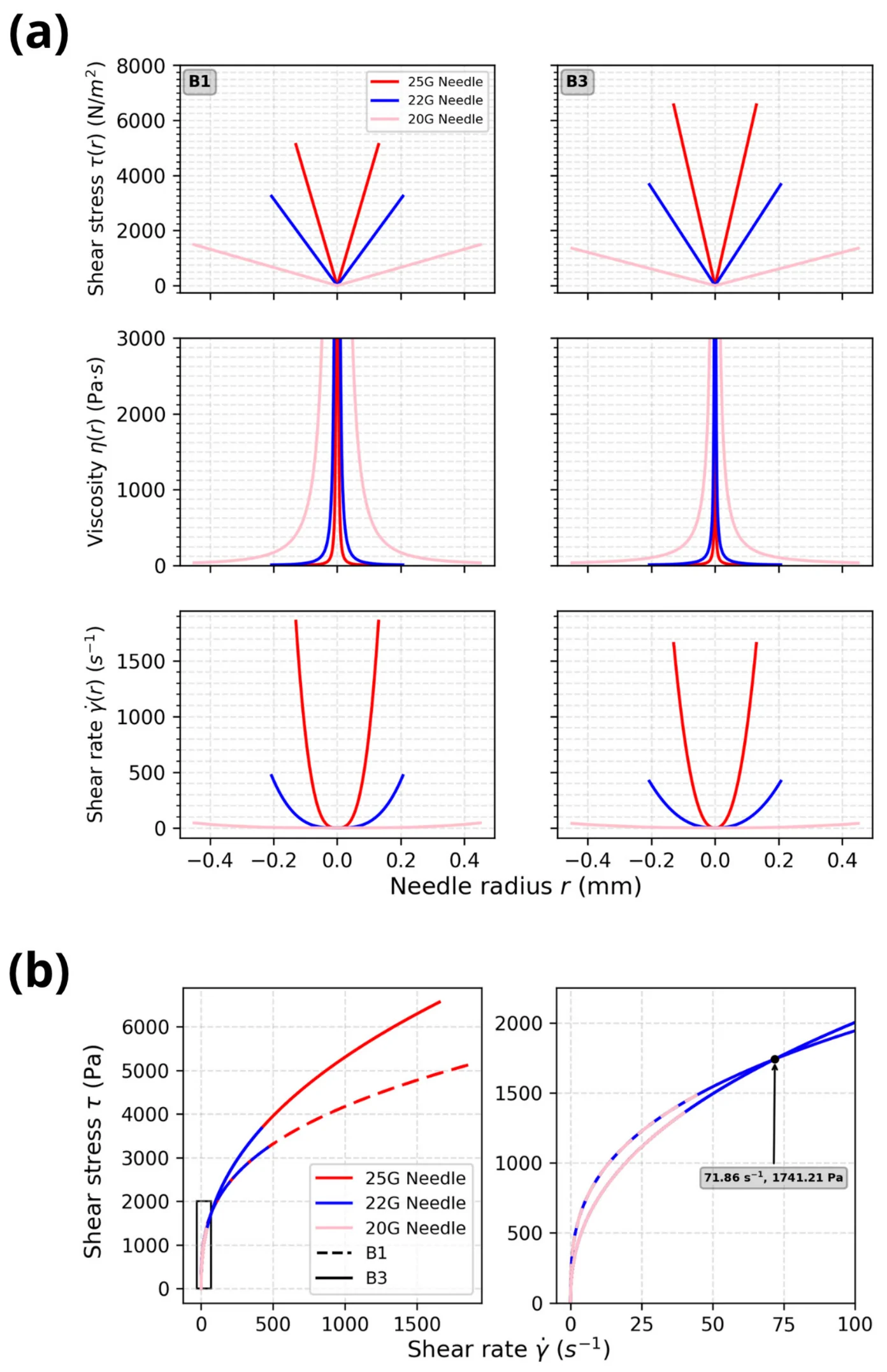
(**a**) Shear stress, viscosity and shear rate profiles for bioinks B1 and B3 when extruded through 20, 22 and 25 G needles. (**b**) Shear stress versus shear rate along the same three needles for bioinks B1 and B3; The right figure is an enlargement of the rectangular region highlighted in the left plot.

The bioink composition and resulting viscoelastic properties also determine the width of the tip that can be used for the bioprinting process [56]. Figure 7a shows the shear stress, viscosity and shear rate profiles for the 20, 22 and 25 G tips. Cells experienced higher shear stress for the 25 G and 22 G needles in B3 than in B1. However, the opposite was observed for the 20 G needle. This apparent contradiction can be understood by examining the viscosity and shear-rate profiles. In the viscosity profiles, it is observed that the viscosity decreases much more rapidly across the needle radius in the case of B3, which is a direct consequence of the smaller power law index of B1 in comparison to B3. However, the shear rate achieved at the needle walls was generally much higher in B1, although this difference between inks decreased as the needle gauge decreased. This reduction led to the shear rate difference in the 20 G needle becoming small enough that the quicker decrease in viscosity in B3 compensated for it, resulting in a lower shear stress for B3 in the 20 G needle than for B1.

This fact can be difficult to visualize in Figure 7a, so a plot of shear stress vs. shear rate is shown in Figure 7b where this difference becomes more evident. The superior performance of B1 was only obvious for higher needle gauges. However, when examining the region corresponding to the 20 G needle, B3 exhibited better performance throughout this tip and even for small radii within the 22 G needle. However, B1 exhibited better performance with larger-gauge needles owing to its lower pseudoplasticity index n, whereas ink B3 performed better with smaller-gauge needles owing to its lower consistency index K.

**Figure 8:**
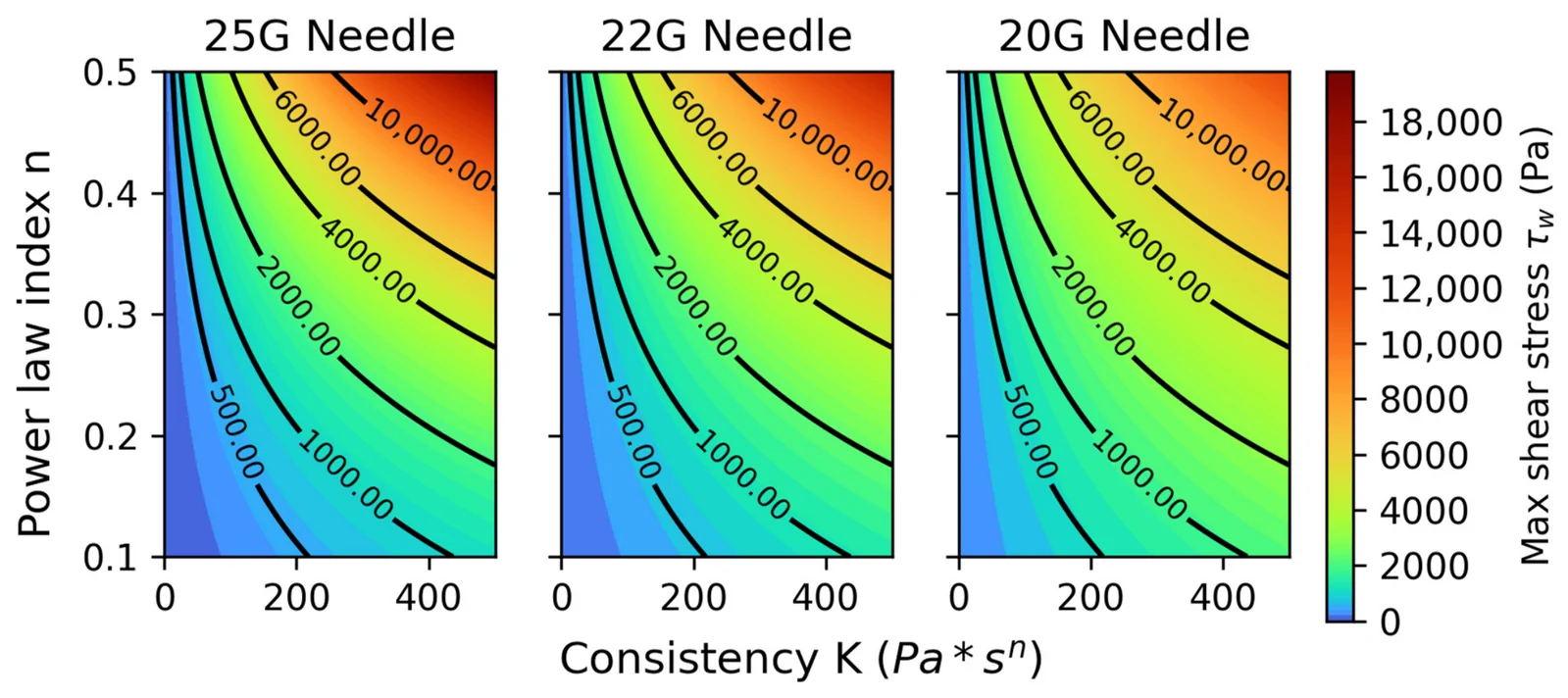
Heatmap of the shear stress as a function of the consistency index *K* and the pseudoplasticity index *n*.

Following the model proposed in [3], a heatmap of the shear stress as a function of the consistency index K and the pseudoplasticity index n was generated and is displayed in Figure 8. From this map, it can be deduced that the minimization of both K and n is the key to ensuring the minimum shear stress experienced by the cells at the tip and then ensuring their survival through the process. Depending on the needle gauge to be used, some flexibility in their magnitude may be allowed; however, taking the 25 G needle, the most restrictive of the analyzed cases, as a reference and the parameters reported by Lemarie et al. [57] for fibroblasts, K should remain below 400 Pa·s^n^ while n should remain below 0.4 to ensure adequate cell viability.

It is of utmost importance to find a relationship between printability and these two indices in order to optimize the design of application-specific bioinks that combine high-fidelity constructs with high cell survival and functionality.

In summary, this study shows that incorporating Sr-OHAp particles and poloxamer F188 into alginate-based bioinks effectively modulates rheological properties to reduce shear stress during bioprinting, thereby improving cell viability. The bioactive nature of Sr-OHAp should further enhance cellular responses, offering a promising strategy for designing bioinks that balance mechanical performance and biological functionality for tissue engineering or modeling applications.

## 5. Conclusions

The incorporation of particles into bioink compositions significantly reduced the consistency index K, whereas the pseudoplasticity index n remained largely unchanged compared to the particle-free compositions. This contrasts with particle-free bioinks containing poloxamer, where K and n were barely modified by the presence of cells, indicating that particles act as sensitizers, specifically affecting consistency without substantially altering the pseudoplasticity. The pseudoplasticity index n also determines the gauge of the tip that can be used with a particular bioink to build application-specific constructs. The K and n indices govern the shear stress experienced by cells during the printing process and, consequently, cell viability. Thus, their relevance extends beyond printing fidelity, becoming fundamental parameters for cell viability in 3D bioprinted constructs.

This work aims to establish a starting point for the transition, in bioink design, from the usual trial-and-error approach toward a more rational, reproducible one. Reporting at least the K and n indices should therefore become standard practice in bioprinting studies, as it is essential for consistent knowledge accumulation across the field.

## Figures and Tables

**Table 1:** Inks and bioinks formulations assayed.

	B1	I2_a,b,c_	B3	I4_a,b,c_
**Sodium alginate (wt%)**	3	3	3	3
**Methyl cellulose (wt%)**	5	5	5	5
**Sr-OHAp (wt%)**	-	-	3	3
**Poloxamer (wt%)**	1.25	1.25	1.25	1.25
**PANC-1 (cells/mL)**	5 × 10^5^	5 × 10^5^	5 × 10^5^	5 × 10^5^

**I2** and **I4_a,b,c_**: a: cells seeded after printing; b: cell free; c: cell and poloxamer free.

## Data Availability

Data are available at https://digital.csic.es/, http://hdl.handle.net/10261/413621 accessed on 11 August 2026.

## Abbreviations

The following abbreviations are used in this manuscript:

SA	Alginic acid sodium salt
MC	Methylcellulose
Sr-OHAp	Strontium enriched hydroxyapatite
I	Ink (cell free extruded)
B	Bioink (cell containing ink extruded)
